# Trends of physical fitness related to weight status: An analysis including over 412,000 Swiss young male conscripts from 2007 to 2022

**DOI:** 10.1016/j.pmedr.2024.102591

**Published:** 2024-01-03

**Authors:** Cédric Gubelmann, Zeno Stanga, Kaspar Staub, Pedro Marques-Vidal

**Affiliations:** a Department of Medicine, Internal Medicine, Lausanne University Hospital (CHUV) and University of Lausanne, Lausanne, Switzerland; b Conscription Centre, Swiss Armed Forces, Payerne, Switzerland; c Medicol Centre Orthopédique d'Ouchy, Lausanne, Switzerland; d Centre of Competence for Military and Disaster Medicine, Swiss Armed Forces, Bern, Switzerland; e Division of Diabetes, Endocrinology, Nutritional Medicine and Metabolism, University Hospital and University of Bern, Bern, Switzerland; f Institute of Evolutionary Medicine, University of Zurich, Zurich, Switzerland

**Keywords:** Physical fitness, Obesity, Weight status, Army, Conscription, Trend, Epidemiology

## Abstract

**Objective:**

The high prevalence of obesity among young adults in the civilian population pose challenges in recruiting physically fit soldiers. We assessed the trend of physical fitness related to weight status among Swiss male conscripts.

**Methods:**

Cross-sectional data of medical examination data during mandatory conscription for the Swiss Armed Forces, 2007–2022 (N = 412,186). The conscription physical test (CPT) assessed five aspects of physical fitness, each aspect scoring 0–25, one component being an endurance test (ET). CPT and ET categories were defined as per military guidelines: “Insufficient”, “Sufficient”, “Good”, “Very Good” and “Excellent”. Weight status was based on body mass index (BMI).

**Results:**

Conscripts with obesity (BMI ≥ 30 kg/m^2^) and overweight (BMI 25–29.99) had significantly (p < 0.001) lower CPT and ET scores compared to normal weight [multivariable-adjusted mean: 54.7 ± 0.1 and 66.5 ± 0.1, *vs.* 73.6 ± 0.1 for CPT; 8.8 ± 0.1 and 12.5 ± 0.1, *vs.* 15.3 ± 0.1 for ET] and a higher likelihood to be categorized as “Insufficient” [weighted relative-risk ratio and (95 %CI): 70.4 (63.7–77.7) and 2.35 (2.16–2.55) for CPT; 77.1 (71.0–83.7) and 3.05 (2.91–3.20) for ET] or “Sufficient” [7.67 (7.38–7.97) and 2.02 (1.99–2.06) for CPT; 8.93 (8.37–9.52) and 2.02 (1.98–2.06) for ET]. Compared to normal weight conscripts, the CPT and ET scores decreased over the conscription years for conscripts with obesity (multivariable-adjusted mean yearly change: −0.11 ± 0.02 for CPT; −0.032 ± 0.007 for ET) and overweight (−0.16 ± 0.01 for CPT and −0.044 ± 0.004 for ET).

**Conclusion:**

Male Swiss conscripts with overweight and obesity have lower physical fitness than normal weight conscripts, and this condition tends to worsen over the conscription years.

## Highlights

• The temporal change in physical fitness related to weight status has never been studied.
• This cross-sectional study includes more than 412,000 Swiss young male over a 15-year conscription period.
• Individuals with obesity and overweight had significantly lower physical fitness compared to those with a normal weight.
• This association appears to worsen over the conscription years, particularly in the musculoskeletal function.
• Lifestyle interventions should prioritize overweight or obese individuals, emphasizing on improving musculoskeletal fitness.

## 1 Introduction

Obesity has emerged as a critical global public health issue, with its prevalence surging in recent decades. According to the World Health Organization (WHO), the global obesity prevalence Worldwide obesity has nearly tripled since 1975, with over 650 million adults classified as obese in 2016 (World Health Organization. Key facts on Obesity and Overweight 9 June, 2021). Switzerland, indeed, has witnessed a notable increase in obesity rates, mirroring this global trend. The Swiss Federal Statistical Office reported that between 1992 and 2017 the percentage of people (aged 15 and above) with obesity in Switzerland doubled from 5 % to 11 %, and a further 31 % were overweight. In the past years, these percentages have stabilised at high levels (Office and Survey, 2017). Obesity not only increases the risk of chronic diseases (World Health Organization. Key facts on Obesity and Overweight 9 June, 2021) but also goes alongside with poor physical performance and musculoskeletal disorders.

Armies rely on a physically fit workforce to ensure operational readiness and success. However, the high prevalence of obesity could pose significant challenges for military recruitment and maintenance of an optimal level of physical fitness among soldiers (McLaughlin and Wittert, 2009). Several armies, including the United States and Switzerland, have developed their own specific maximal weight for height limits for enlistment. Recently, several studies described the negative impact of obesity on physical performance among military conscripts (Staub et al., 2018, Wyss et al., 2019) and personnel (Sanderson et al., 2018): however, these studies missed the opportunity to investigate the changes that may have occurred over time, and relied on relatively old data (2016 and before).

To date, little is known on the temporal trends of physical fitness related to weight categories, despite the need to investigate whether today’s population with obesity experiences a more pronounced decline in physical fitness compared to previous generation. This exploration will give a comprehensive understanding of the evolving impact of obesity on physical fitness and inform evidence-based interventions and strategies to improve the overall fitness and health outcomes of individuals.

Therefore, the aim of this cross-sectional study is to assess the trends of the association between weight status and physical fitness in a population-based sample of Swiss young men aged 18–23 years, over a 15-year conscription period.

## 2 Materials and methods

### 2.1 Study population

As described in details elsewhere (Panczak et al., 2014), all Swiss men reaching the age of 19 each year are summoned for conscription in one of six Swiss Armed Forces conscription centres. The medical examination during conscription serves to check the health status and the physical fitness for military service of the conscript. Standard protocols and measurement tools are used in all centres by trained army medical personnel or specifically trained soldiers.

### 2.2 Data

Fully anonymous, individual conscription records for the period of January 2006 to January 2023 were provided by the Swiss Army (Logistikbasis der Armee, LBA San) under contractual agreement. The delivered anonymized data included the year and centre of conscription of the conscripts, their region of living (canton), age group (<19, 19.00–19.99, 20.00–20.99, 21.00–21.99 or >=22.00 years), height, weight, and physical fitness test.

### 2.3 wt status

Body height (cm) and weight (kg) were measured in underwear and barefoot with a stadiometer and regular calibrated scale used in the conscription centres. BMI [weight (kg)/height (m^2^)] was calculated in accordance with WHO guidelines (World Health Organization. Key facts on Obesity and Overweight 9 June, 2021), and categorized as follows: normal weight for 18.5–24.99, overweight 25–29.99, and obesity ≥ 30 kg/m^2^.

### 2.4 Physical fitness

The physical fitness test at conscription (Conscription Physical Test, CPT) assessed the physical performance of Swiss conscripts in five aspects of fitness. This test was previously validated (Wyss et al., 2007) and described in details elsewhere (Wyss et al., 2007, Office 0000). Before the test, the professional and trained personnel instructed the conscripts regarding the fitness test battery and provided them with a standardized warm‐up. Then, the CPT was conducted with: (i) a progressive endurance run test to measure aerobic endurance capacity, conducted according to the protocol developed by Conconi et al. (Conconi et al., 1982) and evaluated using the final running velocity, (ii) a trunk muscle strength test to assess trunk muscle fitness, (iii) a standing long jump to register the muscle power of the lower extremities, (iv) a seated shotput to register the muscle power of the upper extremities, and (iv) a one leg standing test to measure balance. The CPT total was evaluated on a point scale (0–25 per aspect; in total, maximum 125) (Office 0000). In addition, CPT performance was categorized according to military guidelines as follows: insufficient for < 35, sufficient 35–64, good for 65–79, very good for 80–99, and excellent for ≥ 100 points. The endurance test (ET) was also analysed separately, and categorized as follows: insufficient for < 7, sufficient 7–12, good for 13–15, very good for 16–19, and excellent for ≥ 20 points.

### 2.5 *Inclusion and exclusion* criteria

**Fig. 1:**
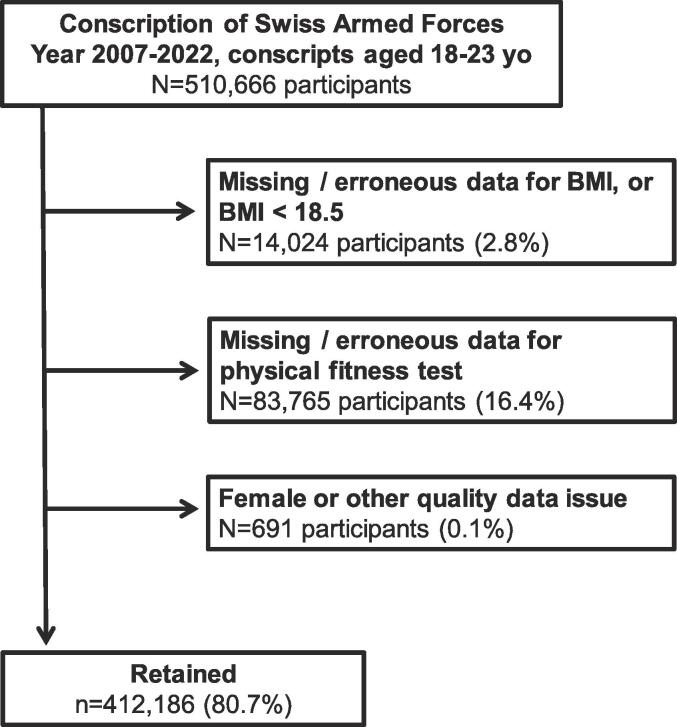
Selection procedure. Conscription of Swiss Armed Forces, 2007–2022.

In this study, only data provided by conscripts aged between 18 and 23 years at conscription were included for analysis. All conscripts younger than 18 and older than 23 years were regarded as not representative, exceptional cases, explained by various reasons as early conscription for air force selection or late conscription after successful naturalization as a Swiss citizen. In addition, participants were excluded if they: (i) had missing data for BMI or a BMI < 18.5 kg/m^2^, or (ii) had missing or erroneous data for physical fitness test, including total CPT and ET, and (iii) were female or having other quality data issues (Fig. 1). We excluded conscripts with a BMI below 18.5 because their condition is likely linked to inherited traits or medical conditions that differ from the lifestyle factors contributing to an elevated BMI.

### 2.6 Ethics

The fully anonymized dataset for this study was provided by the Armed Forces Medical Services (AFMS) on a contractual basis. As described earlier (Panczak et al., 2014), Swiss conscription is mandatory, and the anthropometric measurements used in this study are non-clinical, governmental data. According to the Swiss federal law (‘Bundesgesetz über die militärischen Informationssysteme’ MIG, BG 510.91, Art. 2, 9, 24–29), the Swiss Armed Forces are authorized to make the data accessible for academic research in anonymous form. When dealing with fully anonymized data, no additional ethical approval is needed for analyses based on such governmental data (Swiss Data Privacy Act, SR 235.1; 19.6.1992), which has been further confirmed by the Ethics Committee of Canton Vaud.

### 2.7 Statistical analysis

Statistical analyses were performed using Stata version 16.1 for windows (Stata Corp, College Station, Texas, USA). Descriptive results were expressed as number of participants (percentage) for categorical variables or as average ± standard deviation for continuous variables. Between-group bivariate comparisons were performed using chi-square for categorical variables and student-*t* test and one-way analysis of variance for continuous variables. Multivariable analysis of the CPT and ET scores according to weight categories was performed using analysis of variance. Multivariable analysis of the association between weight and CPT categories was conducted using multinomial (polytomous) logistic regression using the “Good” category as base outcome and normal weight as the reference. Results were expressed as relative risk ratios (RRR, similar to odds ratio) and corresponding 95 % confidence interval. Briefly, a RRR of 2 for overweight participants in the “sufficient” CPT category indicates that, relative to the normal weight group, overweight participants have a doubling of their likelihood to be in the “sufficient” rather than in the “good” category. Multivariable analyses were conducted adjusting for age categories (<19, 19–19.99, 20–20.99, 21–21.99, and > 22 years), region of living (Leman, Mittelland, Northwest, Zurich, Eastern, Central, and Tessin) and conscription period (2007–2011, 2012–2017, and 2018–2022).

As a sizable fraction of the sample was excluded, we performed a sensitivity analysis using inverse probability weighting. Briefly, logistic regression was used to estimate the likelihood of being included for each participant using covariates that were significantly different between included and excluded participants. The inverse of the predicted probability was then used for the analysis of the associations between weight and CPT categories.

A second sensitivity analysis was conducted to assess the association between BMI and the CPT and ET scores as continuous variables. First, Spearman nonparametric correlation was computed; second, a multivariable regression analysis was conducted adjusting on year, age group, and region, and results were expressed as standardized beta coefficients. Finally, we created restricted cubic splines for BMI using the mkspline option of Stata; the position of the knots were assessed using the default settings. We then assessed the graphical association between the predicted values of fitness levels and BMI. For this, CPT and ET were regressed on the resulting BMI splines, and the line associating the predicted values with BMI was plotted.

Due to the large sample size, statistical significance was considered for a two-sided test with p < 0.001.

## 3 Results

### 3.1 *Selection* procedure *and characteristics of the sample*

The selection procedure is indicated in Fig. 1**.** Of the initial 510′666 participants, 412′186 (80.7 %) were retained. Included and excluded participants’ characteristics are presented in Supplementary Table 1. Included participants were younger, more prone to live in Northwest, Eastern or Central Switzerland and to have been recruited in 2018–2022 than the excluded ones. Included participants had a lower prevalence of obesity, had higher CPT and ET scores and were more prone to be in “Good”, “Very Good” and “Excellent” CPT and ET categories.

The characteristics of included participants stratified by weight status are summarized in Table 1. In comparison to participants with normal BMI, participants with overweight and obesity were older, more prone to live in Mittelland and Northwest regions of Switzerland, and to have been conscripted in 2018–2022; they had also lower CPT and ET scores and were more prone to be in “Insufficient” and “Sufficient” CPT and ET categories, this trend being more marked for participants with obesity.

**Table 1:** Characteristics of participants, stratified by weight status. Conscription of Swiss Armed Forces, 2007–2022.

	*Normal weight*	*Overweight*	*Obesity*	P-value
Sample size (Total %)	308,769 (74.9)	84,224 (20.4)	19,193 (4.7)	
Age categories (years)				<0.001
<19	89,023 (28.8)	19,045 (22.6)	4,234 (22.1)	
19–19.99	134,235 (43.5)	35,636 (42.3)	8,082 (42.1)	
20–20.99	56,138 (18.2)	17,391 (20.7)	3,984 (20.8)	
21–21.99	18,050 (5.9)	6,513 (7.7)	1,539 (8.0)	
>22	11,322 (3.7)	5,638 (6.7)	1,354 (7.1)	
Region of living				<0.001
Leman	53,205 (17.3)	13,964 (16.6)	3,306 (17.3)	
Mittelland	67,085 (21.8)	18,384 (21.9)	4,298 (22.4)	
Northwest	50,067 (16.2)	15,174 (18.1)	3,740 (19.5)	
Zurich	46,649 (15.1)	12,484 (14.9)	2,590 (13.5)	
Eastern	46,139 (15.0)	11,807 (14.1)	2,517 (13.1)	
Central	34,265 (11.1)	9,371 (11.2)	2,073 (10.8)	
Tessin	10,944 (3.6)	2.861 (3.4)	630 (3.3)	
Conscription period				<0.001
2007–2011	113,830 (36.9)	30,242 (35.9)	6,451 (33.6)	
2012–2017	134,313 (43.5)	36,925 (43.8)	8,302 (43.3)	
2018–2022	60,626 (19.6)	17,057 (20.3)	4,440 (23.1)	
Conscription Physical Test	73.7 ± 13.5	66.3 ± 14.5	54.4 ± 12.7	<0.001
Categories of CPT				<0.001
Insufficient	1,710 (0.6)	1,216 (1.4)	1,064 (5.5)	
Sufficient	71,434 (23.1)	36,469 (43.3)	13,975 (72.8)	
Good	120,399 (39.0)	29,684 (35.2)	3565 (18.6)	
Very good	109,480 (35.5)	16,041 (19.1)	583 (3.0)	
Excellent	5,746 (1.9)	814 (1.0)	6 (0.03)	
Endurance test	15.3 ± 4.2	12.4 ± 3.9	8.7 ± 3.0	<0.001
Categories of ET				<0.001
Insufficient	4,759 (1.5)	4,228 (5.0)	4,726 (24.6)	
Sufficient	74,523 (24.1)	40,804 (48.5)	12,457 (64.9)	
Good	73,570 (23.8)	19,748 (23.5)	1,593 (8.3)	
Very good	101,489 (32.9)	15,364 (18.2)	390 (2.0)	
Excellent	54,428 (17.6)	4,080 (4.8)	27 (0.1)	

CPT, Conscription Physical Test. ET, Endurance Test. Results expressed as mean ± standard deviation for continuous variables and as number of participants (column percentage) for categorical variables. Between-group comparisons performed by one-way analysis of variance for continuous variables and by chi-square for categorical variables, comparing by weight status.

The multivariable analyses of the associations between weight status and physical tests were described in Table 2 (for CPT) and Table 3 (for ET). Relative to participants with normal weight, participants with overweight and obesity had a higher likelihood to be in “Insufficient” or “Sufficient” CPT and ET categories and a lower likelihood to be in the “Very good” or “Excellent” CPT and ET categories, rather than in the “Good” category. Similar findings were obtained after inverse probability weighting.

**Table 2:** Multivariable analysis of the associations between weight status and conscription physical test. Conscription of Swiss Armed Forces, 2007–2022.

	*Normal weight*	*Overweight*	*P-value*	*Obesity*	P-value
Sample size	308,769	84,224		19,193	
CPT	73.6 ± 0.1	66.5 ± 0.1		54.7 ± 0.1	<0.001§
Categories					
Insufficient	1 (reference)	2.81 (2.61–––3.03)	<0.001	20.7 (19.0–––22.6)	<0.001
Sufficient	1 (reference)	2.05 (2.01–––2.08)	<0.001	6.57 (6.33–––6.83)	<0.001
Very good	1 (reference)	0.60 (0.59–––0.62)	<0.001	0.18 (0.17–––0.20)	<0.001
Excellent	1 (reference)	0.58 (0.54–––0.63)	<0.001	0.03 (0.02–––0.08)	<0.001
Categories †					
Insufficient	1 (reference)	2.35 (2.16–––2.55)	<0.001	70.4 (63.7–––77.7)	<0.001
Sufficient	1 (reference)	2.02 (1.99–––2.06)	<0.001	7.67 (7.38–––7.97)	<0.001
Very good	1 (reference)	0.60 (0.59–––0.62)	<0.001	0.17 (0.16–––0.19)	<0.001
Excellent	1 (reference)	0.58 (0.54–––0.63)	<0.001	0.03 (0.01–––0.07)	<0.001

CPT, Conscription Physical Test. §, for the overall test; †, analysis using inverse probability weighting. Results for continuous variables are presented as multivariable-adjusted mean ± standard error. Results for categories are expressed as relative risk ratio and (95 % confidence interval) using the “Good” category of the conscription physical test as reference and normal weight as comparison group. Statistical analysis by analysis of variance for continuous variables and by multinomial (polytomous) logistic regression for categorical variables. Multivariable analyses were conducted adjusting for age categories (<19, 19–19.99, 20–20.99, 21–21.99, and > 22 years), region of living (Leman, Mittelland, Northwest, Zurich, Eastern, Central, and Tessin) and conscription period (2007–2011, 2012–2017, and 2018–2022).

**Table 3:** Multivariable analysis of the associations between weight status and endurance test. Conscription of Swiss Armed Forces, 2007–2022.

	*Normal weight*	*Overweight*	*P-value*	*Obesity*	P-value
Sample size	308,769	84,224		19,193	
ET	15.3 ± 0.1	12.5 ± 0.1		8.8 ± 0.1	<0.001§
Categories					
Insufficient	1 (reference)	3.23 (3.09–––3.38)	<0.001	46.7 (43.8–––49.8)	<0.001
Sufficient	1 (reference)	2.03 (1.99–––2.07)	<0.001	7.92 (7.51–––8.35)	<0.001
Very good	1 (reference)	0.57 (0.56–––0.58)	<0.001	0.18 (0.16–––0.20)	<0.001
Excellent	1 (reference)	0.28 (0.27–––0.29)	<0.001	0.02 (0.02–––0.03)	<0.001
Categories †					
Insufficient	1 (reference)	3.05 (2.91–––3.20)	<0.001	77.1 (71.0–––83.7)	<0.001
Sufficient	1 (reference)	2.02 (1.98–––2.06)	<0.001	8.93 (8.37–––9.52)	<0.001
Very good	1 (reference)	0.57 (0.56–––0.58)	<0.001	0.16 (0.15–––0.19)	<0.001
Excellent	1 (reference)	0.28 (0.27–––0.29)	<0.001	0.02 (0.01–––0.03)	<0.001

ET, Endurance Test. §, for the overall test; †, analysis using inverse probability weighting. Results for continuous variables are presented as multivariable-adjusted mean ± standard error. Results for categories are expressed as relative risk ratio and (95 % confidence interval) using the “Good” category of the endurance test as reference and normal weight as comparison group. Statistical analysis by analysis of variance for continuous variables and by multinomial (polytomous) logistic regression for categorical variables. Multivariable analyses were conducted adjusting for age categories (<19, 19–19.99, 20–20.99, 21–21.99, and > 22 years), region of living (Leman, Mittelland, Northwest, Zurich, Eastern, Central, and Tessin) and conscription period (2007–2011, 2012–2017, and 2018–2022).

Correlation analysis showed that BMI was inversely associated with CPT and ET (Spearman r = -0.219 and −0.313 for CPT and ET, respectively, both p < 0.001). After multivariable adjustment, the standardized beta coefficients were −0.285 and −0.366 for CPT and ET, respectively, both p < 0.001. Using BMI as restricted cubic splines showed that the association between BMI and CPT and ET had an inverted U-shape, the association increasing from BMI = 18.5 to 22 kg/m2, to decrease afterwards (Supplementary Figs. 1 and 2).

### 3.2 *Trends of* physical *tests related to weight status*

**Fig. 2:**
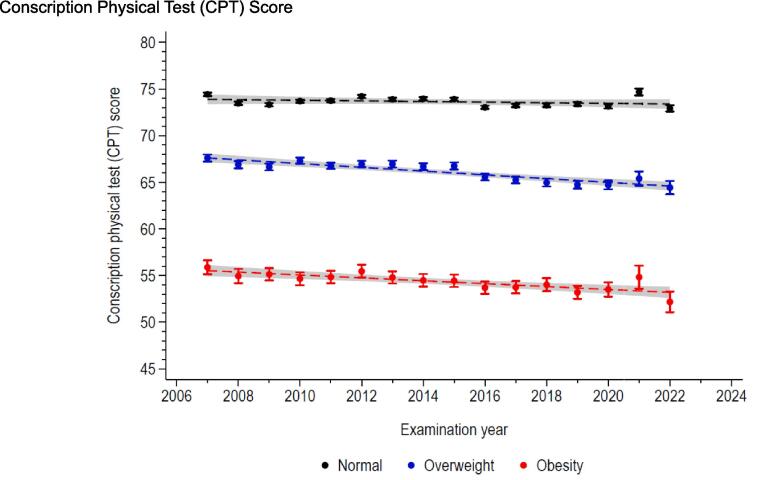
Trends in conscription physical test score, by weight status. Conscription of Swiss Armed Forces, 2007–2022.

**Fig. 3:**
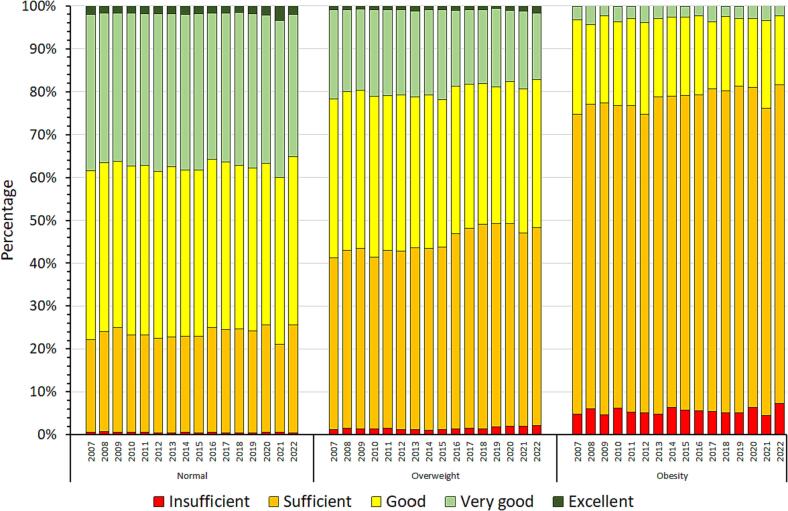
Trends in conscription physical test categories, by weight status. Conscription of Swiss Armed Forces, 2007–2022.

The CPT trends related to weight status are described in Fig. 2 (for scores) and Fig. 3 (for categories). While for normal weight participants the mean CPT score did not significantly change between 2007 and 2022 (less than 1 point), it decreased by an average of 3 points for both participants with overweight and obesity (p for interaction < 0.001, Fig. 2). Similarly, as shown in Fig. 3, the proportions of participants in different CPT categories remained constant over the conscription years for normal weight participants, while the proportion of “Sufficient” increased to the detriment of the “Very good” among participants with overweight and obesity in 2007–2022.

**Fig. 4:**
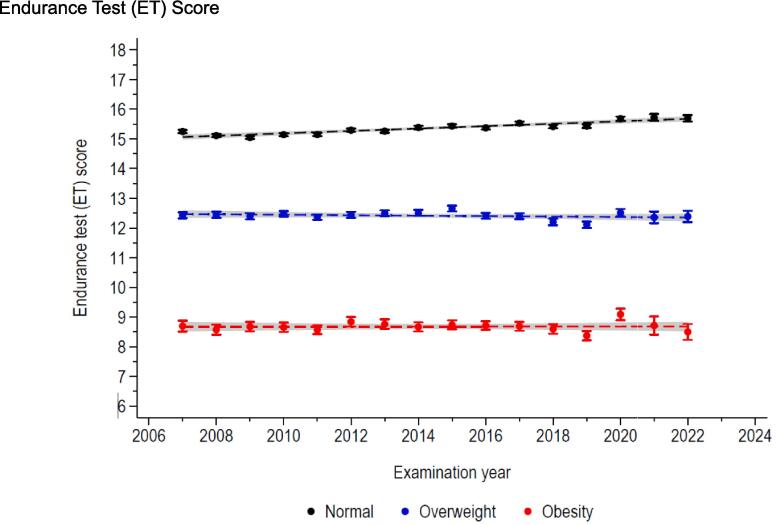
Trends in endurance test score, by weight status. Conscription of Swiss Armed Forces, 2007–2022.

**Fig. 5:**
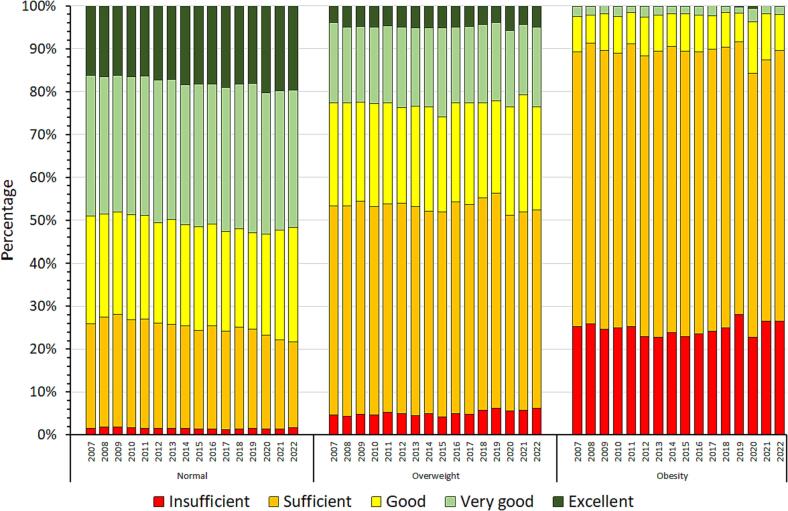
Trends endurance test categories, by weight status. Conscription of Swiss Armed Forces, 2007–2022.

The ET trends related to weight status are described in Fig. 4 (for scores) and Fig. 5 (for categories). While for normal weight participants the mean ET score slightly increased by around one point between 2007 and 2022, it did not vary for both participants with overweight and obesity (Fig. 4). Similarly, for participants with normal BMI, the proportion of “Very good” increased to the detriment of the “Sufficient”, while the ET categories distribution remained constant over the conscription years for participants with overweight and obesity.

The multivariable analysis of the trends between weight status and conscription physical tests are presented in Table 4 and expressed as mean changes per year in CPT and ET scores. Compared to normal weight participants, participants with overweight and obesity decreased their CPT and ET scores.

**Table 4:** Multivariable analysis of the trend of conscription physical tests according to weight status. Conscription of Swiss Armed Forces, 2007–2022.

	*Overweight*	*P-value*	*Obesity*	P-value
Conscription Physical Test	−0.16 ± 0.01	<0.001	−0.11 ± 0.02	<0.001
Endurance Test	−0.044 ± 0.004	<0.001	−0.032 ± 0.007	<0.001

Results are expressed as mean change per year in physical test scores ± standard error relative to the “normal weight” group. Statistical analysis conducted by analysis of variance adjusting for age categories (<19, 19–19.99, 20–20.99, 21–21.99, and > 22 years), region of living (Leman, Mittelland, Northwest, Zurich, Eastern, Central, and Tessin).

## 4 Discussion

This study showed that Swiss male conscripts with overweight and obesity are associated with a consistent decrease in physical fitness, and this association tends to worsen over the conscription years.

### 4.1 *Association* between *weight status and physical tests*

Our findings provide compelling evidence of a strong association between weight status and physical fitness among Swiss young male conscripts. Individuals with obesity and overweight exhibited significantly lower levels of physical fitness compared to those with normal weight. These results highlight that excessive weight not only adversely affects the cardiorespiratory fitness (measured by endurance) but also has a negative impact on the musculoskeletal components, including speed and strength of legs and arms, global trunk strength and coordination. Importantly, this association remained robust even after adjusting for potential confounders such as age, region of living, and conscription year, which are known to influence levels of physical activity and sport participation within the Swiss population (Office fédérale du Sport (OFSPO). Sport Suisse, 2020). Finally, BMI showed a negative association with CPT and ET. The results of the restricted cubic splines analysis showed that the association had an inverted U-shape, the fitness levels increasing slightly till a BMI of approximately 22 kg/m2 to decrease afterwards. This decrease was linear, suggesting that the higher the BMI, the lower the fitness, with no evidence of a threshold.

Several observational studies have consistently demonstrated an inverse relationship between weight status and physical fitness, particularly among children and teenagers (de Lima et al., 2022, Fernandez et al., 2017). Notably, the military environment has provided researchers with a unique opportunity to validate these observations using large samples of young adults (Staub et al., 2018, Wyss et al., 2019, Sanderson et al., 2018). The significance of investigating this relationship within the military lies in the exposure of the body to extreme physiological demands, which necessitate weight restrictions during recruitment to ensure the safety and optimal performance of personnel.

In a study conducted among 1,500 Swiss male conscripts in 2016 (Staub et al., 2018), researchers observed a comparable decrease in both Conscription Physical Test (CPT) and Endurance Test (ET) scores across different BMI categories than our study. This finding was further supported by another study involving a larger sample of Swiss conscripts, which confirmed the negative association between BMI and physical fitness (Wyss et al., 2019). Similarly, studies conducted in the British Armed Forces revealed similar outcomes, where a significant proportion of army personnel with obesity either failed or did not participate in the physical fitness test during their mandatory annual assessment (Sanderson et al., 2018). Finally, one noteworthy contrast between our study and previous military research lies in the substantial magnitude of the effect observed. Specifically, the association between weight status and physical fitness in our study exhibited extreme values, with obese conscripts being over 77 times more likely to fail their physical test.

This finding emphasizes the considerable influence of obesity on physical performance in the context of military conscription in Switzerland. It highlights well the fact that excess weight imposes additional stress on the cardiorespiratory and musculoskeletal system, resulting in diminished strength, endurance, and overall physical performance. The presence of excess adipose tissue in obesity can contribute to reduced mobility and heightened exertion needed to carry out physical tasks effectively.

### 4.2 *Trend* of *physical tests according to weight status*

Our cross-sectional study also investigated the trends in physical fitness performance from 2007 to 2022 considering weight status. Interestingly, we observed a consistent decline in physical fitness among conscripts who were classified as obese or overweight over the conscription years. It is worth noting that this decline was not primarily driven by the cardiorespiratory component (endurance) of physical fitness but rather by the musculoskeletal one. These findings suggest that the negative impact of excess weight on physical fitness may be worsening over time. To the best of our knowledge, no previous study has specifically analysed the trend of physical fitness based on weight status. Although a prior study on Swiss male conscripts reported no changes in physical fitness until 2015, it did not stratify the analysis according to weight status (Wyss et al., 2019).

The decline in physical fitness observed among Swiss male conscripts with excess weight over the conscription years cannot be attributed to higher BMI, as it remained stable in this population (Meili et al., 2022). Moreover, physical activity levels have been reported to increase in the past decade in the Swiss population (Office and Survey, 2017). Instead, it is possible that modern lifestyle factors, such as sedentary behaviour (i.e., mainly time spent sitting), and unhealthy dietary habits contribute to the detrimental impact of excess weight on physical fitness. In particular, the influence of COVID pandemic seems to be excluded as (i) the trend was continuous since 2007, and (ii) the lifestyle changes during the pandemic in this population seems to have been modest (Meili et al., 2022). Also, the reason why the musculoskeletal component appears more prone to worsen over time is not fully understood but might be explained by the non-negligeable genetic influence on cardiorespiratory fitness (Zadro et al., 2017). Nevertheless, further research is required to better elucidate the underlying mechanisms and validate those hypotheses.

### 4.3 *Public health* perspective *for military*

The strong association between obesity and low physical fitness, which tends to worsen over time among conscripts classified as obese or overweight, has significant implications for the recruitment and maintenance of physically fit military personnel. In a military context, optimal physical fitness is essential for successfully completing demanding tasks and missions. Poor physical fitness not only compromises individual performance but also poses a threat to the overall operational readiness of military units. Therefore, targeted lifestyle interventions should prioritize individuals with overweight or obesity, focusing also on improving their musculoskeletal physical fitness, including muscle strength, power, coordination.

## 5 Strengths and limitations

As far as we know, this is the first study investigating the trends of the association between weight status and physical fitness in military conscripts. Importantly, our study included a large sample size of over 412,000 Swiss young men over 15 years and the use of standardized conscription physical tests that assess various aspects of physical fitness.

However, our study also has limitations that should be considered. Firstly, the cross-sectional design of our study precludes the assessment of any causal effect between excess weight and physical fitness. Second, we cannot fully exclude the possibility that despite efforts to standardize data collection procedures, inherent differences may persist across different conscription centres, leading to variations in the measurement of weight or physical fitness. Thirdly, since BMI was the sole indicator of weight status, we could not rule out that some athletes, who may have a high body weight due to higher muscle mass, have been classified as overweight. This could have potentially underestimated the magnitude of the association of overweight with physical fitness in our study; nevertheless, in the absence of other complementary markers, BMI and related WHO categorization remains the most adequate measurement due to its well-established correlation with health outcomes. Fourthly, it's worth noting that a portion of conscripts did not undergo physical fitness assessments. This can be attributed, in part, to the presence of acute or chronic health conditions when they presented at the conscription centre. However, it's important to emphasize that in our dataset, the absence of physical fitness tests only accounted for a relatively small percentage, as illustrated in Fig. 1. Fifth, due to government measures introduced during the COVID pandemic, temporary organizational modifications were required within the conscription centres. These changes resulted in adaptations to the conduct of the physical fitness tests, which may potentially explain the slightly elevated CPT and ET scores observed in 2020–21. Sixth, the available data did not allow us to conduct a comprehensive assessment of the socio-economic level of the conscripts, therefore preventing the adjustment for this parameter. Finally, the study population consists of young men, and the findings may not be generalizable to other populations without Swiss citizenship, other age groups, and women.

## 6 Conclusion

In conclusion, our study shows a strong negative association between excess weight and physical fitness among Swiss male conscripts. Individuals with obesity and overweight demonstrated lower physical fitness scores compared to those with normal weight and this association appears to worsen over the conscription years, particularly for the musculoskeletal dimension.

## Funding

This research did not receive any specific grant from funding agencies in the public, commercial, or not-for-profit sectors.

## 8 Authors’ contribution

CG was partially involved in data collection as a contracted physician for Swiss Armed Forces working at one of the conscription centres, made part of the statistical analyses and wrote most of the article; ZS and KS contributed to the revisions of the paper, has read and approved the final version of the manuscript; PMV made part of the statistical analysis, wrote part of the article and revised the article for important intellectual content.

## 9 Data access

The data are generated and owned by the Swiss Armed Forces. The data and the permission to use them are available from the Swiss Armed Forces (Logistikbasis der Armee-LBA San) on submission and approval of a study protocol.

## CRediT authorship contribution statement

**Cédric Gubelmann:** Conceptualization, Formal analysis, Investigation, Methodology, Project administration, Resources, Software, Validation, Visualization, Writing – original draft, Writing – review & editing. **Zeno Stanga:** Conceptualization, Project administration, Resources, Supervision, Validation, Writing – review & editing. **Kaspar Staub:** Data curation, Formal analysis, Validation, Writing – review & editing. **Pedro Marques-Vidal:** Conceptualization, Formal analysis, Investigation, Methodology, Project administration, Resources, Software, Supervision, Validation, Visualization, Writing – original draft, Writing – review & editing.

## Declaration of competing interest

The authors declare that they have no known competing financial interests or personal relationships that could have appeared to influence the work reported in this paper.

## Data Availability

The authors do not have permission to share data.
