# Supplementary material for: Trends of physical fitness related to weight status: An analysis including over 412,000 Swiss young male conscripts from 2007 to 2022
Source: Prev Med Rep. 2024 Jan 3;38:102591. doi: 10.1016/j.pmedr.2024.102591 (PMC10809177; doi:10.1016/j.pmedr.2024.102591)
Supplement: Supplementary data 1 [file mmc1.docx]

# Supplementary tables

**Supplementary table 1:** Characteristics of excluded and included participants. Conscription of Swiss Armed Forces, 2007-2022.

|  | ***Included*** | ***Excluded*** | **P-value** |
| --- | --- | --- | --- |
| Sample size (Total %) | 412,186 (80.7) | 98,480 (19.3) |  |
| Age categories (years) |  |  | <0.001 |
| <19 | 112,302 (27.3) | 15,020 (15.3) |  |
| 19-19.99 | 177,953 (43.2) | 33,712 (34.2) |  |
| 20-20.99 | 77,513 (18.8) | 23,032 (23.4) |  |
| 21-21.99 | 26,102 (6.3) | 11,148 (11.3) |  |
| >22 | 18,314 (4.4) | 15,565 (15.8) |  |
| Region of living |  |  | <0.001 |
| Leman | 70,475 (17.1) | 19,516 (20.0) |  |
| Mittelland | 89,767 (21.8) | 25,279 (26.0) |  |
| Northwest | 68,981 (16.8) | 11,340 (11.6) |  |
| Zurich | 61,723 (15.0) | 18,123 (18.6) |  |
| Eastern | 60,463 (14.7) | 11,646 (12.0) |  |
| Central | 45,709 (11.1) | 6697 (6.9) |  |
| Tessin | 14435 (3.5) | 4800 (4.9) |  |
| Conscription period |  |  | <0.001 |
| 2007-2011 | 150,523 (36.5) | 38,536 (39.1) |  |
| 2012-2017 | 179,540 (43.6) | 44,182 (44.9) |  |
| 2018-2022 | 82,123 (19.9) | 15,761 (16.0) |  |
| BMI categories |  |  | <0.001 |
| Normal | 308,769 (74.9) | 61,387 (64.4) |  |
| Overweight | 84,224 (20.4) | 19,718 (20.7) |  |
| Obesity | 19,193 (4.7) | 14,242 (14.9) |  |
| Conscription Physical Test | 71.3 ± 14.5 | 37.1 ± 29.6 | <0.001 |
| Categories of CPT |  |  | <0.001 |
| Insufficient | 3990 (1.0) | 7605 (40.6) |  |
| Sufficient | 121,878 (29.6) | 7333 (39.1) |  |
| Good | 153,648 (37.3) | 2374 (12.7) |  |
| Very good | 126,104 (30.6) | 1361 (7.3) |  |
| Excellent | 6566 (1.6) | 79 (0.4) |  |
| Endurance test | 14.4 ± 4.4 | 9.6 ± 5.6 | <0.001 |
| Categories of ET |  |  | <0.001 |
| Insufficient | 13,713 (3.3) | 4568 (36.6) |  |
| Sufficient | 127,784 (31.0) | 4005 (32.1) |  |
| Good | 94,911 (23.0) | 1658 (13.3) |  |
| Very good | 117,243 (28.4) | 1524 (12.2) |  |
| Excellent | 58,535 (14.2) | 724 (5.8) |  |

BMI, Body Mass Index. ET, Endurance Test. CPT, Conscription Physical Test. Results expressed as mean ± standard deviation for continuous variables and as number of participants (column percentage) for categorical variables. Between-group comparisons performed by Student t-test for continuous variables and by chi-square for categorical variables, comparing included and excluded participants.
