# Supplementary figures and images for: Trends of physical fitness related to weight status: An analysis including over 412,000 Swiss young male conscripts from 2007 to 2022
Source: Prev Med Rep. 2024 Jan 3;38:102591. doi: 10.1016/j.pmedr.2024.102591 (PMC10809177; doi:10.1016/j.pmedr.2024.102591)

## Slide 1
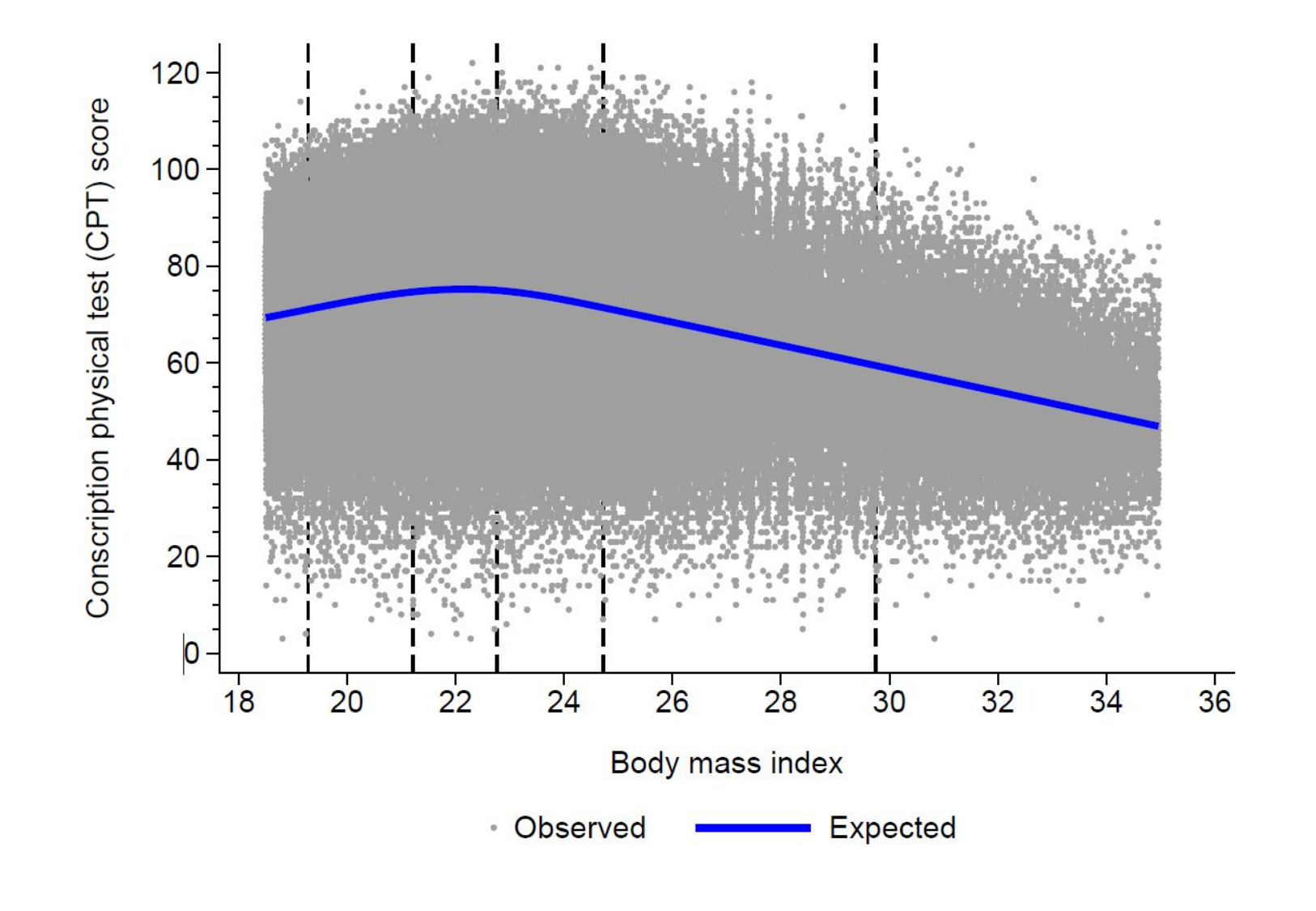

Supplement: Supplementary data 2 [file mmc2.pptx]

## Slide 1
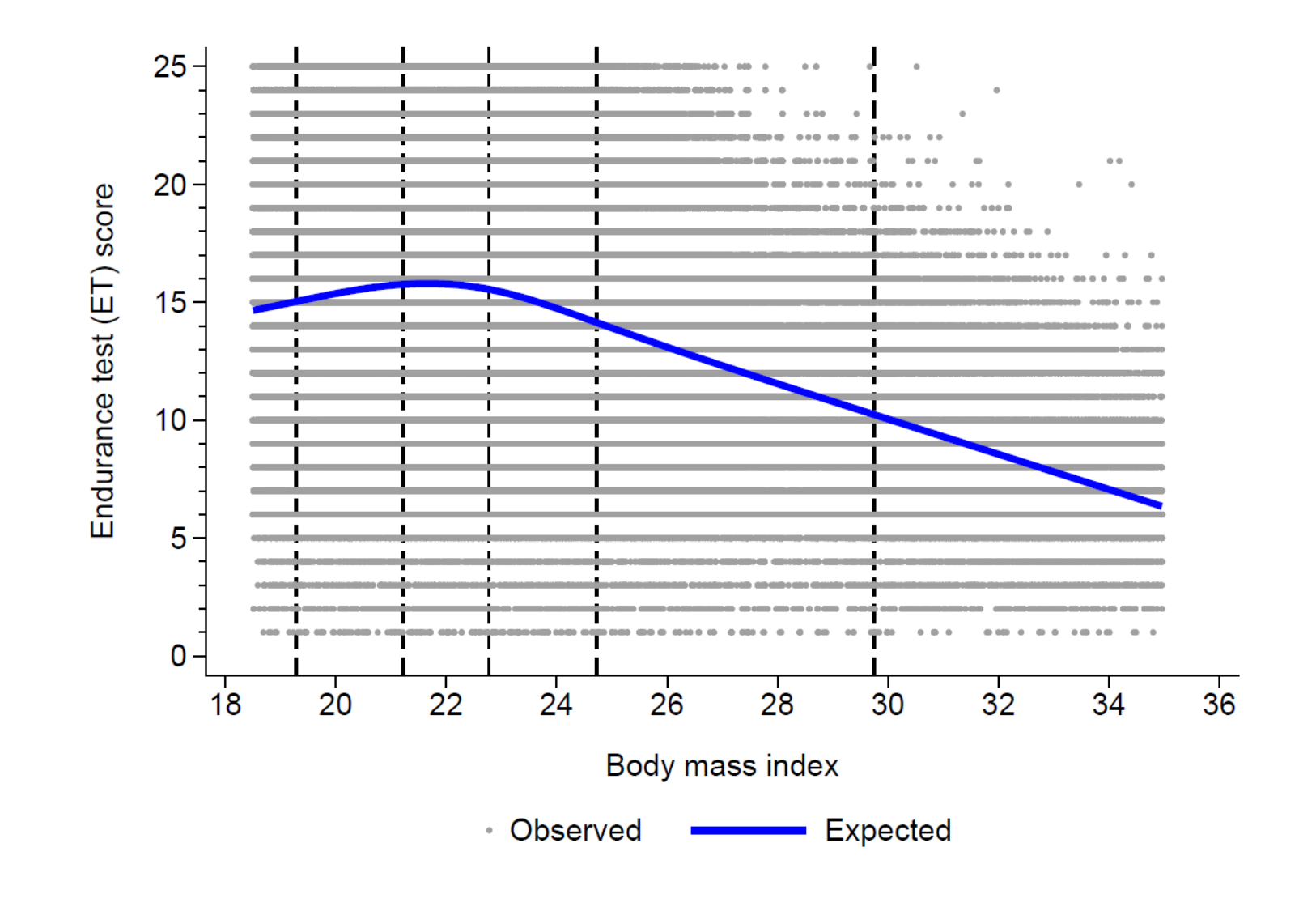

Supplement: Supplementary data 3 [file mmc3.pptx]
